# Perceptions and experiences of nurses on the care of children with malnutrition under 5 years at Intermediate Hospital Katutura, Khomas region, Namibia

**DOI:** 10.4102/hsag.v31i0.3355

**Published:** 2026-07-20

**Authors:** Ndamononghenda Malangu, Hans J. Amukugo, Salomo Salomo

**Affiliations:** 1 School of Nursing and Public Health, Faculty of Health Sciences and Veterinary Medicine, University of Namibia, Windhoek, Namibia

**Keywords:** perceptions, experiences, nurses, care, malnutrition, children

## Abstract

**Background:**

Malnutrition in children under 5 years commonly presents as stunting, wasting or micronutrient deficiencies. These conditions lead to high admission rates and nursing burnout at Intermediate Hospital Katutura, Khomas region, Namibia. Despite this impact, a significant knowledge gap exists regarding the perceptions and experiences of Namibian nurses in caring for these children.

**Aim:**

This study explored and described the perceptions and experiences of nurses regarding the care of malnourished children under five in the paediatric ward.

**Setting:**

The study was conducted in the paediatric ward of Intermediate Hospital Katutura, Khomas region, Namibia.

**Methods:**

A qualitative, exploratory, descriptive and phenomenological design was employed. Data were collected from 11 nurses via purposive sampling through individual face-to-face interviews and analysed according to themes. Trustworthiness and ethical principles were rigorously applied.

**Results:**

Five themes emerged: (1) patient and caregiver-related factors; (2) experiences of health facilities or institution-related factors; (3) perceived health provider-related challenges; (4) health provider perceptions of facilitators and recommendations; and (5) perceived legal factors.

**Conclusion:**

Nurses identified significant barriers to care, including a lack of specialised training, negative workplace relationships and systemic socio-economic challenges like poverty and gender-based violence. Issues with health worker relationships and the availability of childcare acts and guidelines were also identified. Therefore, the Ministry of Health and Social Services needs to recruit additional staff and ensure sufficient resources.

**Contribution:**

This study identifies the need for specialised postgraduate paediatric training, providing a framework for the development of malnutrition management guidelines and establishing an evidence base for nurse-led community awareness programs.

## Introduction

Malnutrition is defined by the World Health Organization (WHO) (2024) as any deficiency, excess or imbalance in an individual’s intake of energy and nutrients. Singh (2019:4) defines malnutrition as an ‘insufficient intake of energy and nutrients’. This condition negatively affects normal bodily functions, metabolic processes, physical activity levels and overall growth and development in children. Addressing these challenges is essential, as improving the quality of paediatric malnutrition care can generate significant social benefits by enhancing long-term health outcomes and reducing the socio-economic burden on communities (UNICEF 2023:12).

Malnourished children worldwide are managed through community-based management of acute malnutrition (CMAM) strategies, which enable community healthcare workers to identify affected children early and initiate treatment before their condition becomes severe (Govender et al. 2021:450). Ready-to-use therapeutic food and other nutrient-dense forms are included in the community-based strategies, which facilitate the early detection and management of severe acute malnutrition in cases without medical complications (Govender et al. 2021:452). These clinical interventions are complemented by primary health care initiatives integrated into the road-to-health booklet system, such as exclusive breastfeeding, immunisation and optimal complementary feeding (Govender et al. 2021:40).

A wide range of factors negatively impact the provision of care for children admitted with malnutrition. Globally, nurses report that unreasonable policies, insufficient staffing and excessive administrative burdens hinder effective care, often leading to diminished morale and a lack of support (Buckley et al. 2020:210; Tamata et al. 2021:1201). In various international contexts, these challenges are compounded by limited career progression, financial constraints and the denial of leave because of chronic staff shortages (Moradi et al. 2021:562).

Despite these global insights, there is a significant knowledge gap regarding the specific experiences of nurses within the Namibian public health sector (Shifiona & Ashipala 2023:31). Specifically, it remains unclear how the unique socio-economic landscape and regional malnutrition trends shape the perceptions of nurses at Intermediate Hospital Katutura. Exploring these experiences in the local context is essential to develop culturally and institutionally relevant interventions that improve paediatric outcomes and support the nursing workforce (Ministry of Health and Social Services [MoHSS] 2021:15). The purpose of this study was to describe the perceptions and experiences of nurses about the care of malnourished children at Intermediate Hospital Katutura (IHK) in the Khomas region, Namibia.

### Study objectives

The objectives of the study were to:

- Explore the perceptions and experiences of nurses on the care of children with malnutrition under 5 years in paediatric wards at IHK, Khomas region, Namibia.
- Describe the perceptions and experiences of nurses on the care of children with malnutrition under 5 years in paediatric wards at IHK, Khomas region, Namibia.

### Study purpose

The purpose of the study was to explore and describe the perceptions and experiences of nurses regarding the care of children with malnutrition under 5 years at IHK, Khomas region, Namibia.

## Theoretical and conceptual framework

For this study, the socio-ecological model was used as a guiding framework. Its specific application will be discussed in more detail below.

### Socio-ecological model

The socio-ecological model, initially conceived by Urie Bronfenbrenner in the 1970s as a conceptual model for understanding human development, was subsequently formalised into a theory in the 1980s (Kilanowski 2017:103). This foundational theory is illustrated by a series of nesting circles, positioning the individual at the centre, enveloped by various interconnected systems: the patient (or individual), microsystem, mesosystem, exosystem and chronosystem.

Each of these systems will be discussed in further detail here.

#### Patient

The patient is considered the central focus of the nesting circles (Kilanowski 2017:104). A patient is defined as a distinct individual who requires medical treatment (Oben 2020:4). In this study, ‘patient’ refers to the children under the age of 5 years admitted with malnutrition. In the context of this study, this human connection is vital as nurses at Intermediate Hospital Katutura navigate the complex emotional and physical needs of malnourished children and their caregivers (Oben 2020:5).

#### Microsystem

The microsystem, being the closest to the patient, exerts the strongest influences and encompasses the interactions and relationships within their immediate surroundings (Kilanowski 2017:104). Microsystems are the interpersonal factors that affect the patients’ care, such as relationships between health workers and patients, as well as the relationship between health workers and caregivers or parents of children with malnutrition. The microsystem of the paediatric ward functions as a relational hub where the delivery of healthcare services is inseparable from the empathy and compassion the health professional holds for those seeking assistance (Oben 2020:8).

#### Mesosystem

The mesosystem refers to the environment in which the patient stays, the religious beliefs and the relationship between the nurses, doctors, and caregivers or parents, as all these factors can either positively or negatively affect or influence the care of patients.

#### Exosystem

Kilanowski (2017:15) explains that while the exosystem does not have a direct impact on the patient, it nonetheless exerts both detrimental and beneficial interactive forces upon them. For this study, the exosystem encompasses health facility-related factors, including available resources, staffing arrangements and in-service training aimed at upskilling and educating the healthcare professionals who provide patient care to malnourished children.

#### Chronosystem

The chronosystem covers both the internal and external aspects of time, as well as historical context (Kilanowski 2017:105). In this study, the chronosystem relates to the legal factors that can influence the care of malnourished children, such as policies, guidelines and cultural beliefs and norms of patients.

## Research methods and design

### Study setting

The researcher purposively selected IHK, a major Namibian public referral hospital specialising in paediatric malnutrition. Eleven male and female nurses from both day and night shifts were interviewed to provide diverse clinical perspectives.

### Design

This study adopted a qualitative approach, which enabled the researcher to explore the depth, richness and complexity inherent in the lives of nurses caring for malnourished children (Grove, Burns & Gray 2013). This study employed an exploratory, descriptive and phenomenological contextual design to gain a comprehensive understanding of the lived experiences of nurses caring for malnourished children. This approach refers to a research method that explores and offers a deeper understanding of practical problems (Tenny et al. 2022:15). By utilising this design, the researcher was able to uncover hidden patterns, explore diverse viewpoints, and develop a more profound understanding of complex social issues (Tenny et al. 2022:15). Specifically, this qualitative design enabled the researcher to explore the depth, richness and complexity inherent in the lives of nurses at Intermediate Hospital Katutura as they manage the challenges associated with paediatric malnutrition (Grove et al. 2013:201).

#### Exploratory design

In this study, an exploratory design was adopted, which enabled the researcher to explore the lived experiences and perceptions of nurses caring for malnourished children.

#### Descriptive design

This study described perceptions and experiences of nurses caring for malnourished children.

#### Phenomenological design

A phenomenological design was adopted to deeply explore and describe the lived experiences of nurses caring for children with malnutrition, aiming to understand the meaning of their perceptions and experiences.

### Context

This study was conducted at the paediatric ward of IHK. A paediatric ward was selected because malnourished children under the age of 5 years are nursed there.

### Study population and sampling strategy

The study population consisted of 800 enrolled and registered nurses working at IHK. The nurses at IHK were selected for this study because they represent an information-rich population with the direct clinical experience necessary to address the research objectives. The researcher therefore ensured that the data gathered were grounded in the actual daily realities of paediatric malnutrition care, thereby pinpointing the specific characteristics and professional insights required for a deep understanding of the phenomenon (Momoh 2022:45).

In this study, the researcher adopted non-probability purposive sampling, where participants are deliberately chosen based on their specific knowledge of the phenomena (Brink, Van der Walt & Van Rensburg 2018:160; Grove, Gray & Burns 2015:250). This method is considered the most effective way to gain an in-depth understanding and discover meaning in complex clinical experiences (Grove et al. 2015).

The participants consisted of eleven nursing professionals, comprising 10 Registered Nurses and one Enrolled Nurse. These specific categories were selected to ensure a comprehensive perspective of the care provided, as Registered Nurses often handle clinical management and policy implementation, while Enrolled Nurses provide direct, continuous bedside care to malnourished children.

The final sample size was determined by data saturation, which occurs when additional sampling provides redundant information rather than new insights (Grove et al. 2015:252). For inclusion, the nurses had to meet specific inclusion criteria: they must have worked in the paediatric ward for at least 1 year and expressed a willingness to participate. These criteria ensure that participants possess the necessary professional characteristics to be considered part of the target population (Grove et al. 2013).

### Data collection

Following recruitment, which involved the researcher personally visiting the paediatric wards at IHK to distribute information leaflets and obtain written informed consent, individual face-to-face interviews were conducted. These interviews were held in a private room within the ward to ensure confidentiality and were conducted in English, as it is the official language of professional communication in Namibian healthcare.

To ensure the gathering of in-depth data, the researcher utilised an interview guide, an audio recorder and field notes (Polit & Beck 2017; Sharma 2022). The interview guide provided a consistent framework of open-ended questions, while the audio recorder captured the participants’ spoken accounts for accurate verbatim transcription (Creswell 2014:130). To complement the recordings, field notes were used to document non-verbal cues and descriptive accounts of the interactions (Phillippi & Lauderdale 2018:118). Other measures to ensure depth included the use of probing, paraphrasing and clarification during the interviews. A primary challenge encountered was the busy schedule of the nurses, which was managed by scheduling interviews during overlap shifts or quieter periods to avoid disrupting patient care.

### Data analysis

Data analysis in this study followed a systematic thematic approach to transform raw verbal accounts into meaningful findings. Once interviews were completed, the audio recordings were captured and transcribed verbatim by the researcher. To ensure data cleaning and accuracy, the transcripts were cross-checked against the original audio files multiple times to correct any discrepancies and ensure that non-verbal cues from the field notes were integrated where relevant (Polit & Beck 2017).

The researcher implemented the five-phase coding process (Bigham & Witkowskey 2022:30) to provide a structured framework for analysis. The application of these phases in the study was as follows:

- Phase 1: Familiarisation and Preparation: They repeatedly read the transcripts and reviewed field notes to gain a holistic sense of the nurses’ experiences.
- Phase 2: Initial Coding: Using the similarity principle, textual data were broken down into smaller units and assigned labels or codes to represent recurring content and symbols (Brink et al. 2018; Polit & Beck 2017:160).
- Phase 3: Generating Subthemes: The initial codes were grouped based on shared concepts. These subthemes acted as specific elements under a broader umbrella, such as grouping codes related to staff shortages and lack of equipment (Caulfield 2019:12).
- Phase 4: Developing and Reviewing Themes: Subthemes were synthesised into five major themes that represented recurring patterns across the entire dataset, such as health facility-related factors (Table 1).
- Phase 5: Finalising and Naming: The researcher refined the themes to address the research objective regarding nurses’ perceptions and experiences of malnutrition care at IHK.

**TABLE 1:** Overview of research themes and evidence from participant interviews.

Themes	Subthemes	Condensed participant quotes	Demographics
1. Patients and parents or caregivers-related factors and challenges	1.1. Parents’ or caregivers’ attitude	‘When parents have a good … attitude … everything flows smoothly. But when they become … disrespectful … this negatively affects the child.’	P-F, M, 27
	1.2. Cultural beliefs, norms and practices	‘Some parents prefer … herbalists … they tend to go to witchdoctors … this then worsens their condition.’	P-A, F, 31
	1.3. Inadequate knowledge	‘Lack of knowledge can be a cause of malnutrition, because even if some of the parents/caregivers of malnourished children have enough food of different varieties, most of them do not know how they are supposed to feed their children or prepare this food.’	P-B, F, 27
2. Experiences of socio-economic challenges	2.1. Socio-economic hardships	‘Unemployment causes malnutrition cases to rise among children under 5 years as these parents cannot afford proper food for their children.’	P-F, M, 27
		‘… they are unable to afford proper meals such as a healthy, balanced diet.’	P-D, F, 34
	2.2. Alcohol and drug abuse	‘Some parents are into alcohol and drug abuse, they spend most of their money on alcohol rather than spending money on food …’	P-K, F, 26
	2.3. Gender-based violence	‘… if the mother has an abusive partner … they end up neglecting these kids, this then leads to malnutrition because there is nobody to cook …’	P-J, F, 25
	2.4. Ignorance	‘Another thing is just pure ignorance … the child does not want to eat, and they just let it be, they do not really go the extra mile …’	P-J, F, 25
3. Experiences of institution-related barriers	3.1. Lack of human resources	‘Challenges include … the shortage of staff … managing plus-minus seventy patients with malnutrition.’	P-A, F, 31
	3.2. Shortage of infrastructure	‘… lack of medication … leading to these children being treated maybe with second-line antibiotics which can also lead to drug resistance.’	P-J, F, 25
	3.3. Broken and malfunctioning of equipment	‘Everything … is broken, including cot beds; mommies are sleeping on the floor … which is very unhygienic.’	P-C, F, 32
	3.4. Lack of financial resources	‘… when you are ordering milk … it is out of stock … all because of lack of financial resources.’	P-I, F, 27
4. Perceived health provider-related challenges	4.1. Burnout among nurses	‘It is really overwhelming, it is a lot of work for one person, so that leads to burnout.’	P-G, M, 28
	4.2. Inadequate knowledge on the management of malnutrition	‘Some nurses … might even have forgotten … they might stick to the routine work and forget … the correct way of doing things.’	P-H, M, 29
5. Health providers’ perceptions of facilitators and recommendations	5.1. Interprofessional and caregiver relationships	‘When there is a good relationship between the nurse and the doctor [*it*] will also help in the quality of care …’	P-J, F, 25
	5.2. Professional ethical behaviour	‘The nurses should work together … as this will improve the quality of nursing care.’	P-H, M, 29

This thematic approach was chosen for its flexibility, allowing the researcher to focus on the deep meanings inherent in the complex social issue of paediatric malnutrition care (Caulfield 2019; Creswell 2014).

### Measures to ensure trustworthiness

The researcher adopted the criteria of credibility, dependability, confirmability, transferability and authenticity to ensure the study’s robustness (Grove et al. 2015):

- *Credibility*: Was ensured through prolonged engagement, with the researcher spending 2 months in the field to build rapport and reach data saturation. Member checking was conducted by replaying audio recordings to participants to verify their responses. The findings were subjected to peer review by a master’s graduate and supervisor audits (Polit & Beck 2018).
- *Dependability*: The researcher provided a dense description of the methodology and used triangulation by collecting data from various participants using an interview guide, audio recorder and field notes. An independent audit of the coding process was performed to ensure consistency (Brink et al. 2018).
- *Confirmability*: Objectivity was maintained through an audit trail, where an external expert scrutinised the research process, and the supervisor mentored the researcher to ensure findings were grounded in the data rather than researcher bias (Korstjens & Moser 2018).
- *Transferability*: Achieved by providing a dense description of the research context and utilising purposive sampling, allowing readers to determine the applicability of the findings to similar paediatric settings (Ahmed 2024).
- *Authenticity*: The researcher ensured a faithful representation of the participants’ realities by transcribing all audio verbatim, documenting non-verbal gestures in field notes, and utilising direct quotations in the report (Polit & Beck 2017).

### Ethical considerations

Ethical clearance to conduct this study was obtained from the University of Namibia Ethics Committee on 02 November 2023. The ethical clearance number is DEC OSH 0083. Permission from the MoHSS and the Regional Director of Khomas region (reference number of 22/4/3) was acquired. The objectives of the research were explained to the participants, and written informed consent was obtained. The participants were not asked for their names; instead, they were all coded to ensure confidentiality and protect their privacy. This ethical safeguard was applied to uphold the principle of Beneficence. The study adhered to four core principles:

- *Respect for persons*: Participants were treated as autonomous agents with the right to self-determination. Written informed consent was obtained after explaining the study objectives, and participants were informed of their right to withdraw without penalty (Brink et al. 2018:170). To ensure anonymity and privacy, participants were identified via codes rather than names, and data access was limited to the research team.
- *Beneficence*: The researcher maximised benefits while ensuring non-maleficence by protecting participants from psychological or social harm (Grove et al. 2015). Participants could decline any questions that caused discomfort.
- *Justice*: Was upheld through the equitable selection of participants; all nurses meeting the inclusion criteria had an equal opportunity to participate and were treated with consistency using a standardised interview guide (Polit & Beck 2017:175).
- *Scientific honesty*: Integrity was maintained through ensuring all sources were accurately acknowledged, peer review by a postgraduate and supervisor review.

## Results

### Theme 1: Patients and parents or caregivers-related factors and challenges

This theme identifies nursing challenges arising from the behaviours and knowledge gaps of those responsible for the children. While the children are the patients, the clinical barriers are primarily driven by caregiver attitudes and non-compliance.

#### Subtheme 1.1: Parents’ or caregivers’ attitude

Negative or uncooperative attitudes from caregivers hinder collaboration and care delivery. Participants indicated that a lack of respect or a refusal to follow instructions directly impacts the child’s progress:

‘When parents have a good and a positive attitude towards the nurses caring for their children, then everything flows smoothly. But when they become very difficult and disrespectful towards the nurses this negatively affects the condition of the child.’ (P-F, M, 27)

#### Subtheme 1.2: Cultural beliefs, norms and practices

Cultural beliefs significantly influence care-seeking behaviours, as parents often prioritise traditional or spiritual remedies over hospital-based care. Participants noted that these cultural norms frequently delay medical intervention:

‘Some parents prefer taking their children to herbalists … they tend to go to witchdoctors to have [*traditional procedures performed*] … this then worsens their condition.’ (P-A, F, 31)

#### Subtheme 1.3: Inadequate knowledge

Participants highlighted a critical disconnect between the availability of resources and the knowledge required to utilise them effectively. This deficit is fuelled by a lack of visible clinical directives and educational outreach. Findings suggest that even when caregivers have access to a variety of food, a lack of guidance on preparation and evidence-based feeding practices hinders nutritional recovery and child outcomes:

‘Lack of knowledge can be a cause of malnutrition, because even if some of the parents or caregivers of malnourished children have enough food of different varieties, most of them do not know how they are supposed to feed their children or prepare this food.’ (P-B, F, 27)

### Theme 2: Experiences of socio-economic challenges

This theme highlights the wider social and economic determinants within the community that contribute to the high incidence of childhood malnutrition.

#### Subtheme 2.1: Socio-economic hardships

Participants identified a symbiotic relationship between high unemployment rates and household poverty as the primary structural driver of childhood malnutrition. The lack of stable income directly translates into food insecurity, where families are unable to afford a balanced diet or maintain basic hygiene standards. This financial strain creates a cycle where late hospital presentations become common because of the costs associated with seeking care:

‘Unemployment causes malnutrition cases to rise among children under five years as these parents cannot afford proper food for their children.’ (P-F, M, 27)

#### Subtheme 2.2: Alcohol and drug abuse

Substance abuse by parents or caregivers was reported to lead to child neglect, poor parenting and the misuse of limited household income:

‘Some parents are into alcohol and drug abuse they spend most of their money on alcohol rather than spending money on food to feed their kid to avoid malnutrition.’ (P-K, F, 26)

#### Subtheme 2.3: Gender-based violence

Violence within the household was found to negatively impact maternal mental health and bonding, leading to neglect of feeding responsibilities and poor nutritional outcomes:

‘… if the mother has an abusive partner, most of the time they end up neglecting these kids, this then leads to malnutrition because there is nobody to cook for the kids …’ (P-J, F, 25)

#### Subtheme 2.4: Ignorance

Parental ignorance, described as an intentional lack of attention or simple negligence, contributed to delayed action on warning signs of malnutrition:

‘Another thing is just pure ignorance of these parents, you will find parents that have this kid that has maybe lost appetite but the parent is just like no the child does not want to eat and they just let it be …’ (P-J, F, 25)

### Theme 3: Experiences of institution-related barriers

This theme explores systemic obstacles that hinder quality care. Effective paediatric malnutrition management relies on adequate staffing, functional equipment and consistent supplies, all of which were reported as deficient.

#### Subtheme 3.1: Lack of human resources

Participants identified chronic understaffing as a primary cause of burnout and potential negligence. High patient-to-nurse ratios prevent the delivery of the intensive care required:

‘Challenges include … the shortage of staff … you may find yourself one registered nurse in the ward … managing plus-minus seventy patients with malnutrition.’ (P-A, F, 31)

#### Subtheme 3.2: Shortage of infrastructure

A deficiency in medical supplies and essential equipment, such as intravenous administration controllers, which leads to treatment delays and compromised clinical outcomes:

‘… lack of medication … leading to these children being treated maybe with second-line antibiotics which can also lead to drug resistance.’ (P-J, F, 25)

#### Subtheme 3.3: Broken and malfunctioning of equipment

Non-functional equipment forces nurses to improvise or borrow from other wards, which diminishes patient safety and efficiency:

‘Everything in this hospital is broken, including cot beds; mommies are sleeping on the floor with their sick children which is very unhygienic.’ (P-C, F, 32)

#### Subtheme 3.4: Lack of financial resources

Participants perceived budgetary constraints and a lack of government funding as the root cause of the scarcity of therapeutic milk and medical technology:

‘… when you are ordering milk … it is out of stock … or they are not enough for all the patients and that is really a challenge all because of lack of financial resources.’ (P-I, F, 27)

### Theme 4: Perceived health provider-related challenges

Participants narrated that internal professional struggles, specifically burnout and knowledge deficits, directly hindered their ability to provide quality care. The findings revealed a workforce struggling to maintain clinical standards under extreme pressure.

#### Subtheme 4.1: Burnout among nurses

Nurses expressed that the overwhelming workload leads to physical exhaustion and an inability to provide the specialised attention required:

‘It is really overwhelming, it is a lot of work for one person so that leads to burnout.’ (P-G, M, 28)

#### Subtheme 4.2: Inadequate knowledge on the management of malnutrition

Participants identified a gap in specialised clinical knowledge regarding current malnutrition protocols, often as a result of nurses relying on outdated routines:

‘Some nurses … might even have forgotten some of the knowledge that they gained at school … they might stick to the routine work and forget … the correct way of doing things.’ (P-H, M, 29)

### Theme 5: Health providers’ perceptions of facilitators and recommendations

This theme outlines the factors participants believe are essential for improving outcomes, focusing on interpersonal dynamics and professional ethics.

#### Subtheme 5.1: Interprofessional and caregiver relationships

Participants perceived that the success of a malnutrition care plan depends on collaborative communication between doctors, nurses and the child’s guardians:

‘When there is a good relationship between the nurse and the doctor helps in the quality of care that is rendered to these patients.’ (P-J, F, 25)

#### Subtheme 5.2: Professional ethical behaviour

The findings suggest that non-judgmental and supportive attitudes are vital for ensuring that caregivers adhere to medical treatment plans:

‘The nurses should work together … and have a positive and supportive attitude … as this will improve the quality of nursing care.’ (P-H, M, 29)

## Discussion

The successful management of malnutrition at IHK is heavily dependent on the cooperation and attitudes of those responsible for the child. This study found that negative caregiver attitudes and traditional remedies, such as seeking herbalists because of beliefs in ‘witchcraft’, frequently result in late hospital presentations and severe clinical complications. This creates a ‘dual burden’ for nursing staff who must manage medical emergencies while navigating deep-seated cultural mistrust. These findings are supported by Amado et al. (2023), who note that cultural norms often conflict with hospital-based nutritional protocols, as well as Shapi and Teti (2021), who observed similar patterns in Southern Africa, where traditional medicine is prioritised over clinical intervention. Furthermore, a significant knowledge-to-action gap exists; even when food is available, caregivers often lack the skills to prepare it effectively. This aligns with Elhady et al. (2023) and suggests that clinical recovery is unsustainable without a corresponding community-based education strategy.

Beyond individual factors, environmental root causes such as poverty and unemployment act as primary drivers of food insecurity in informal settlements. However, the findings indicate that malnutrition is a complex social pathology rather than a purely economic one. Participants linked alcohol and drug abuse, gender-based violence (GBV) and general ignorance to pervasive child neglect. Safarani et al. (2018) corroborate this, stating that children in high-stress environments where household income is diverted to substance abuse are at a significantly higher risk of severe acute malnutrition (SAM). The breakdown of the child-caregiver dyad, often catalysed by GBV and substance abuse, remains a critical barrier to long-term nutritional rehabilitation.

Systemic failures within the health facility directly impact clinical safety and the ability to adhere to international standards. Dangerous nurse-to-patient ratios (recorded as 1:85) make the implementation of the WHO 10-step protocol, which requires intensive monitoring during the ‘initial stabilisation’ phase, virtually impossible. This infrastructure deficit is compounded by malfunctioning equipment and a lack of financial resources, specifically a scarcity of intravenous (IV) automatic control machines and therapeutic milks such as F75 and F100. Nakweenda, Anthonie and Van der Heever (2022) found similar resource constraints in Namibian public health sectors, noting that without specialised feeds or IV pumps, nurses cannot safely manage Step 1 (preventing hypoglycaemia) or Step 4 (correcting electrolyte imbalance) of the WHO guidelines. Consequently, nurses are forced into a ‘survival mode’ where evidence-based protocols are sacrificed for basic supervision.

The nursing workforce is currently struggling to maintain clinical standards under this extreme systemic pressure. Burnout was identified as a critical barrier, where physical exhaustion leads to emotional detachment, and care left undone, a phenomenon also emphasised by Takalani et al. (2018). A significant gap in specialised skills was also revealed, as many nurses relied on outdated routines rather than the Integrated Management of Childhood Illness (IMCI) guidelines. This ‘knowledge decay’ suggests that without continuous professional development (CPD) or regular workshops, facilities operate on legacy knowledge that does not account for modern, evidence-based practices.

Despite these challenges, interpersonal and ethical factors remain essential facilitators for improving paediatric outcomes. Participants perceived that success depends on collaborative communication between doctors, nurses and guardians. This finding is central to the clinical microsystem, where the healthcare encounter is a human-to-human connection (Oben 2020). Clinical outcomes reportedly improve when nurses transition from an ‘authoritative’ role to a ‘collaborative’ one. This relational approach suggests that non-judgmental and supportive attitudes are vital for ensuring that caregivers from diverse backgrounds adhere to medical plans. By respecting cultural nuances while teaching modern nutritional methods, professionals honour their ethical duty to provide compassionate care.

Finally, the lack of administrative and legal support serves as a major grievance. The shortage of current policies and the absence of malnutrition-related standing orders leave nurses without point-of-care directives. In the absence of visible IMCI posters or localised guidelines, nurses are left professionally and legally vulnerable when making critical decisions. Furthermore, a deficiency in legal awareness regarding child protection laws contributes to the cycle of neglect. This suggests that healthcare providers may not fully see themselves as ‘legal advocates’ for the child because the legal frameworks meant to protect vulnerable children, such as the *Namibian Constitution* and *Child Care and Protection Act*, are not sufficiently integrated into daily clinical practice.

In summary, the experiences of nurses at IHK reveal that childhood malnutrition is a multi-dimensional crisis where clinical management is constantly undermined by systemic, socioeconomic and educational barriers. The most significant finding of this research is the clear link between institutional resource shortages, specifically staffing and therapeutic supplies and the inability to fulfil international WHO and IMCI protocols. This study contributes to the field by triangulating these clinical failures with socioeconomic drivers such as unemployment and GBV, as well as the critical knowledge deficit regarding both medical guidelines and child protection laws. Ultimately, these findings indicate that improving paediatric outcomes in the Khomas region requires a move away from isolated clinical treatment toward a multi-sectoral approach. This strategy must integrate institutional capacity building with community-based nutritional education and the legal empowerment of both providers and caregivers to break the cycle of malnutrition

### Strengths and limitations

A primary strength of this study was the use of a qualitative descriptive design, which allowed for a rich, detailed exploration of the lived experiences and challenges faced by nurses (Creswell 2014). The use of verbatim transcriptions and member-checking ensured that the findings are a credible reflection of the participants’ realities (Polit & Beck 2017). However, the study is limited by its focus on a single institution, which may affect the transferability of the findings to different healthcare contexts or rural facilities with different resource levels (Brink et al. 2018).

Additionally, as data were collected through face-to-face interviews, social desirability bias may have influenced some participants to provide responses that reflect more positively on their professional conduct (Grove et al. 2015). The study also focused solely on the nursing staff perspectives; excluding the voices of parents, doctors and hospital management limits the breadth of the institutional and social dynamics explored. Finally, the cross-sectional nature of the data collection means that the findings represent a snapshot in time and may not account for seasonal variations in malnutrition rates, which are common in sub-Saharan Africa (Amado et al. 2023).

### Implications and recommendations

The findings of this study have significant implications for health policy, nursing management and professional development within the Namibian healthcare system. To address insufficient human resources and the resulting burnout among nurses, it is recommended that the MoHSS and IHK management conduct formal, transparent workload assessments. These assessments should inform evidence-based staffing augmentation and the establishment of mandatory psychosocial support programs for paediatric staff.

Regarding nursing practice and support, there is an urgent need to prioritise the maintenance and repair of broken or malfunctioning equipment and to secure a consistent supply chain for therapeutic milks (F75 and F100) to ensure clinical safety and protocol adherence. Education and community outreach must be strengthened by implementing regular in-service training to bridge the knowledge gap in malnutrition management. Furthermore, equipping nurses with advanced communication skills is essential to effectively navigate complex cultural beliefs, norms, and practices.

At the policy level, the MoHSS should focus on integrating healthcare services with social welfare programs to address the structural root causes of malnutrition, such as poverty and unemployment.

Finally, for future research, a follow-up qualitative study should be conducted to explore the perceptions of parents and caregivers, as well as hospital management and social workers. Including these diverse role players will provide a more holistic understanding of the care continuum and identify systemic bottlenecks from multiple professional and personal perspectives

## Conclusion

This study explored and described the lived experiences of nurses caring for malnourished children under five at IHK, revealing a professional environment defined by moral distress and systemic limitation. In fulfilment of the first objective to explore these experiences, the findings uncover a complex burden where nurses must navigate not only clinical malnutrition but also the profound socioeconomic vulnerabilities of caregivers, such as poverty and substance abuse. This suggests that the nursing experience at IHK extends far beyond medical treatment, requiring nurses to act as social mediators in the face of late hospital presentations and child neglect. Regarding the second objective to describe these experiences, the study characterises the care process as a struggle against institutional scarcity. Nurses described a significant inability to adhere to the WHO 10-step protocol because of shortages of essential therapeutic milks (F75/F100) and specialised equipment, exacerbated by staffing shortages and a lack of legal guidance on child protection. Collectively, these findings signify that while nurses possess the clinical intent to provide quality care, their experiences are defined by routine rather than specialised practice. Ultimately, the study concludes that improving paediatric outcomes in the Khomas region requires moving beyond clinical guidelines to address the systemic resource gaps and community-level barriers that define the daily reality of nursing staff.
